# Friendly Injury

**Published:** 1852-12

**Authors:** 


*Friendly Injury.*—In the Bee, of this city, the following statement appeared the past week:—"A suit was lately brought in Barnstable county, which grew out of the simple act of shaking hands. The defendant, it appears, seized the hand of the plaintiff to shake it, and in doing so, he grasped it so tightly as to crush the bones and thereby cripple it forever. The hand became ulcerated, and many of the bones have been discharged from the wound. The result of the trial is not yet heard."—*Ibid.*

